# Personal

**Published:** 1894-09

**Authors:** 


### Personal.

---

Dr. F. G. Moehlau, formerly of 1266 Jefferson street, Buffalo, has been appointed superintendent of the Steuben Sanitarium at Hornellsville, N. Y.

---

Dr. Henry Osthues, who graduated at Niagara University in May, and who has received the New York State license to practise medicine, after an examination in which he was classed among the honor men, has located at 1266 Jefferson street, Buffalo, and assumed the practice vacated by Dr. Moehlau, consequent upon his removal to Hornellsville.

---

Dr. Thomas Lothrop, of Buffalo, has returned from his European visit and resumed his practice. He looks much invigorated by his rest, and will resume his obstetric lectures at Niagara University on Tuesday, September 25, 1894.

---

Dr. Swan M. Burnett, of Washington, D. C., who was attending the ophthalmological congress in Edinburgh, was called home on account of the illness of his son Vivian, who is suffering from typhoid fever at the home of his tutor in Washington. Mrs. Frances Hodgson Burnett, who had just opened her house in London, also came in obedience to a similar summons. It will be recalled by lovers of that beautiful story that Vivian, now seventeen years old, is said to be the original "Little Lord Fauntleroy."

---

Dr. Charles Huntoon Knight has removed from 20 West 31st street to 145 West 57th street, New York City. Hours: 9 to 12 and 5 to 7.

---

Dr. William C. Krauss, of the Journal staff, sailed for Europe, August 16, 1894, on the steamer "Columbia," to spend a few weeks in Hamburg and Berlin. He expects to return about the 20th of September.

---

Dr. Richard Henry Satterlee, formerly a member of the surgical staff of the Manhattan Eye and Ear Hospital, of New York, announces to the medical profession that he has opened an office at 189 Delaware avenue, Buffalo. Practice limited to diseases of the eye and ear. Hours, 9 to 1 and by appointment.

---

Dr. C. B. Burr, of Pontiac, has resigned as superintendent of the Eastern Michigan Asylum, a hospital for the insane, to accept an appointment as superintendent of Oak Grove, a private institution for the treatment of the insane, located at Flint, Mich. Oak Grove was built about four years ago, and is said to be one of the finest institutions of its kind in the country. It is situated in a large grove of natural trees and is practically perfect in point of equipment and arrangement.

Dr. Burr's reputation as an alienist is second to none in the country and his ability will have ample scope at Oak Grove. He is succeeded at Pontiac by Dr. E. A. Christian, formerly assistant superintendent of the Eastern Asylum.

---

Dr. Byron Stanton, of Cincinnati, has been appointed president of the Ohio State Board of Health. Dr. Stanton's experience as a sanitarian has been verified by service as health officer of Cincinnati, and his promotion to this important office is a fitting recognition thereof.

---

Dr. E. Arnold Praeger, formerly of Nanaimo, B. C., has removed to Los Angeles, California. His practice is limited to gynecology. His office is at rooms 56-57 in the Potomac Block, Broadway. Hours, 10 to 3.

